# Signal Detection of Depression and Suicidality Associated with Finasteride and Dutasteride: Updated Pharmacovigilance Evidence and Recommendations for Comprehensive Psychiatric Assessment

**DOI:** 10.3390/brainsci16040394

**Published:** 2026-04-04

**Authors:** Stefania Chiappini, John Martin Corkery, Amira Guirguis, Alessio Mosca, Mya Murray, Davide Arillotta, Luigi Dattoli, Giovanni Martinotti, Stefania Bonaccorso, Fabrizio Schifano, Nicolò Schifano

**Affiliations:** 1 School of Medicine, UniCamillus University, via di S. Alessandro 8, 00131 Rome, Italy; 2 Psychopharmacology, Drug Misuse and Novel Psychoactive Substances Research Unit, Hertfordshire Medical School, University of Hertfordshire, Hatfield AL10 9AB, UK; j.corkery@herts.ac.uk (J.M.C.);; 3 Pharmacy, Medical School, Swansea University, Swansea SA2 8PP, UK; amira.guirguis@swansea.ac.uk (A.G.);; 4 Department of Neuroscience, Imaging and Clinical Sciences, “G. D’Annunzio” University, 66100 Chieti, Italy; 5 North Islington Core Team, RCPsych, London N7 8US, UK; 6 ASST Sette Laghi–Circolo e Fondazione Macchi Hospital, via Luigi Borri 57, 21100 Varese, Italy; 7 School of Medicine, Università degli Studi dell’Insubria, via Ravasi 2, 21100 Varese, Italy

**Keywords:** finasteride, dutasteride, FAERS, pharmacovigilance, suicide, depression, anxiety, libido, sexual dysfunctions

## Abstract

**Background/Objectives**: Finasteride and dutasteride are 5α-reductase inhibitors that block the conversion of testosterone to dihydrotestosterone, reducing androgenic stimulation of tissues such as the prostate and hair follicles. Used mainly for benign prostatic hyperplasia and androgenic alopecia, finasteride selectively inhibits type-2 5α-reductase isoenzyme, while dutasteride inhibits both type-1 and type-2. Although sexual adverse effects like erectile dysfunction are well-documented, emerging evidence suggests possible neuropsychiatric reactions—including depression, suicidal ideation, and cognitive decline—potentially linked to reduced neurosteroid synthesis, such as that of allopregnanolone. Causality cannot be inferred from spontaneous reporting data. This study aimed to assess pharmacovigilance signals for psychopathological disorders associated with finasteride and dutasteride in the FAERS database. **Methods**: Cleaned FAERS data referring to years up to 2025 after deduplication were analyzed, excluding non-serious cases and those without the drug as the sole suspect (MedDra 29.0). Reporting Odds Ratios (RORs) with 95% CIs were calculated to compare psychiatric reactions between finasteride and dutasteride. Python 3.11 was used to screen and summarize relevant cases, accounting for differences in total case numbers. **Results**: This pharmacovigilance study analyzed FAERS data to assess the neuropsychiatric and sexual adverse reactions associated with finasteride and dutasteride. Depression, anxiety, suicidality, and libido-related issues were reported more frequently for finasteride, especially in younger men using low-dose therapy for alopecia. Potential mechanisms include reduced neurosteroid synthesis, androgen/sex-hormone axis disruption, altered hippocampal neurogenesis, and dopaminergic changes. **Conclusions**: A baseline psychiatric assessment and the regular monitoring of mood, sexual function, and suicidal ideation are recommended. Limitations include under-reporting, reporting bias, and a lack of incidence data. The findings underscore the need for ongoing surveillance and controlled studies to clarify the clinical significance of these signals.

## 1. Introduction

Finasteride and dutasteride are 5α-reductase inhibitors (5-ARIs) which competitively inhibit the 5-AR isoenzymes, thereby blocking the conversion of testosterone to its more-potent metabolite dihydrotestosterone (DHT) [1]. By reducing DHT levels, 5-ARIs effectively attenuate the androgenic stimulation of a number of target tissues, including the prostate and the hair follicles [2]. The 5-ARIs are used mainly for the treatment of benign prostatic hyperplasia (BPH) and androgenic alopecia (AGA). Two main 5-AR isotypes are represented; whilst the type-1 5-AR isoenzyme is found in most of the tissues of the body, the type-2 isoenzyme is more specific for the genito-urinary tissues [3]. Overall, finasteride is selective for the type-2 isoenzyme, and dutasteride inhibits less selectively both isotypes [4]. Due to their interaction with the 5-AR isoenzymes, the 5-ARIs may be associated with a range of different adverse drug reactions (ADRs), most notably the well-documented sexual adverse effects, such as erectile dysfunction and disturbances of sexual desire [5]. A number of studies identified a possible increased risk of persistent side effects despite discontinuation of the 5-ARIs, an issue which has been associated with a controversial clinical entity known as ‘post-finasteride syndrome’ [6]. These persistent side effects can be mediated by a decreased effect of DHT on the male genitalia and may include penile atrophy and an increased incidence of Peyronie’s disease, although any causal association remain questionable [7]. Although the sexual-domain ADRs are very well documented, a growing body of evidence including pharmacovigilance data [8] and clinical studies [9] has identified a possible association between 5-ARI use and the onset of a spectrum of neuropsychiatric ADRs including depression, suicidal ideation and cognitive decline. A mechanism to explain this possible association is a putative decrease in the synthesis of a range of neurosteroids, including allopregnanolone, which modulate GABAergic neurotransmission and exert antidepressant and neuroprotective effects [10]. Given the lack of comprehensive randomized studies, any causal relationship between 5-ARIs and their psychopathological side effects remains incompletely understood. A comprehensive understanding of this issue is crucial, given the widespread and often long-term use of 5-ARIs in otherwise healthy populations.

Using a pharmacovigilance approach, drug safety may be investigated in a real-world, post-marketing setting. Unlike pre-marketing clinical trials, which are conducted in controlled environments with highly selected populations and limited sample sizes, pharmacovigilance databases allow for the detection of rare, delayed, or unexpected adverse drug reactions in broader and more heterogeneous populations [11].

Aim of the study: The aim of the current analysis was to investigate, using a pharmacovigilance approach, the potential association between reports of various psychopathological disorders in the FDA Adverse Event Reporting System (FAERS) database and the intake of finasteride or dutasteride.

## 2. Materials and Methods

### Data Analysis

Data were extracted from the FAERS dataset due to its accessibility, as it is a publicly available database, allowing for the reproducibility and transparency of analyses. Also, it is one of the largest global pharmacovigilance databases, increasing the likelihood of capturing rare or emerging safety signals. FAERS is widely used in pharmacovigilance research and by regulatory agencies, making the findings more comparable with existing literature [12]. Cleaned case listings were used for the purposes of data analysis; more precisely, after deduplication, cases without the drug of interest as the only suspect active ingredient and cases marked as ‘non-serious’ were excluded (Supplementary Figures S1 and S2). Firstly, the RORs were calculated, comparing psychiatric reactions for finasteride to dutasteride and vice versa according to the Medical Dictionary for Regulatory Activities (MedDRA) (29.0) (see Supplemental Material). To gather the maximum relevant data, Python 3.11 was adopted to write a programme to screen the included case studies and summarize the number of cases involving each psychiatric reaction as listed. The ROR and 95% confidence interval (CI) values were then calculated using Equations (1) and (2) [13] (Table 1). This disproportionality analysis was conducted to account for the large levels of discrepancies between the two drugs’ total case listings (e.g., 4132 cases for finasteride compared to 202 for dutasteride) alone.

(1) $$ROR=\frac{\left(A/B\right)}{\left(C/D\right)}$$

Equation (1): Equation used to calculate the ROR where A–D are the values as identified in Table 1.

(2) $$CI=exp(ln(\vee )\pm 1.96\times \sqrt{\frac{1}{A}+\frac{1}{B}+\frac{1}{C}+\frac{1}{D}})$$

Equation (2): Equation used to calculate 95% Confidence Interval (CI) where A–D are the values as identified in Table 1.

When the ROR > 1 but the CIs were <1, a reaction was deemed not statistically significant and was removed from further discussion. Furthermore, a similar code was written to extract the relevant case listings (e.g., no other suspect active ingredient; serious cases only; one or more reactions of interest reported) to a Microsoft Excel (Version 2509, 2025) spreadsheet to be eventually used to extract demographic data. Finally, a third code was written using Python to extract relevant clinical and demographic information. This included ‘Reason for Use’, ‘Outcome’, ‘Ages’, ‘Country where event occurred’, ‘Reporter Types and ‘Latest FDA received date’. This data was written on a Microsoft Excel spreadsheet for each drug of interest for further analysis and presentation. Case listings were grouped into ‘Anxiety related’, ‘Libido related’, ‘Suicidality related’, and ‘Depressive related’ reactions to allow for a wider range of demographic analysis.

## 3. Results

### 3.1. Descriptive Data

**Figure 1:**
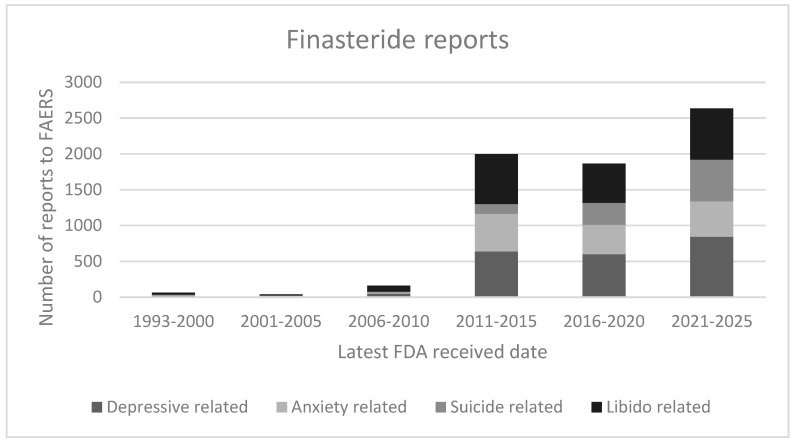
Finasteride reports by year of record.

**Figure 2:**
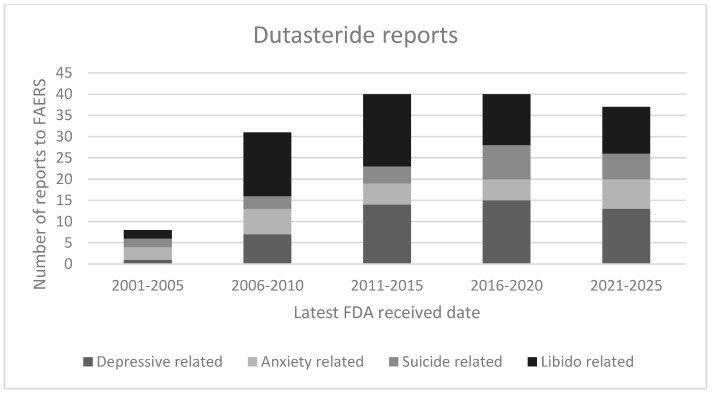
Dutasteride reports by year of record.

According to the latest (e.g., 2025) FAERS dataset, a total number of 4132 finasteride cases were reported. The most frequent psychiatric events included depressive-related ADRs (*n* = 2252), libido disorders (*n* = 2122), anxiety-related ADRs (*n* = 1524), and suicidality (*n* = 1109). Conversely, the total serious psychiatric ADRs reported up to 2025 with dutasteride as the suspect active ingredient were 202 (Table 2). Among the dutasteride-related ADRs, disorders of libido represented the most frequently documented category (*n* = 57), followed closely by depressive symptoms (*n* = 55). Anxiety-related ADRs (*n* = 27) and suicide-related events (*n* = 24) also occurred with notable frequency. Across all ADR categories, adult patients between 19 and 64 years of age represented the majority of cases for both finasteride and dutasteride (Table 2). When the ADRs’ outcomes were provided, most cases were classified as “disabled”, as defined by U.S. reporting regulations, suggesting a substantial impact of these ADRs on the subjects’ functional status [11]. A temporal analysis revealed a progressive rise in the number of reported ADRs over time, with the highest reporting rates observed between 2020 and 2025 (Figure 1 and Figure 2).

Geographically, Great Britain and the United States were the countries with the highest number of submissions across all psychiatric ADR groups for both finasteride and dutasteride.

### 3.2. Disproportionality Analysis

The disproportionality analysis comparing finasteride with dutasteride showed consistently higher values for finasteride across all examined ADR categories. Specifically, finasteride was associated with an elevated likelihood of reporting depression-related ADRs (ROR = 3.20; 95% CI: 2.33–4.39), suicide-related ADRs (ROR = 2.72; 95% CI: 1.77–4.19), libido-related ADRs (ROR = 2.68; 95% CI: 1.96–3.67), and anxiety-related ADRs (ROR = 3.79; 95% CI: 2.51–5.70), as presented in Table 3.

The ROR values were also stratified according to the age (<45 years vs. ≥45 years) and use time trend (every 5 years interval) (Table 4).

## 4. Discussion

To our knowledge, few studies have comparatively examined finasteride- and dutasteride-related depressive, anxiety, and suicide ADRs reported to the FAERS dataset. Overall, these psychopathological issues, together with libido-related ADRs, were found here to be more frequently reported for finasteride compared to dutasteride. This is consistent with previous epidemiological and pharmacovigilance evidence for an association of 5-ARIs (especially finasteride) with mood disorders, depression and self-harm [8,9]. A large observational cohort study found that older men (66 years+) on 5-ARIs had an increased risk of depression and self-harm compared with unexposed men, although no statistically significant increased risk of suicide overall was identified [14]. Consistently, a pharmacovigilance case/non-case study, considering 3282 finasteride cases using VigiBase, found a disproportional signal of suicidality, depression and anxiety associated with finasteride use in younger men (<45 years) treated for alopecia [15]. Similarly, a signal-detection study found 395 suicidality reports and 1299 depression reports in global pharmacovigilance data from more than 140 countries for finasteride and dutasteride [16]. The rising trend in reporting ADRs may reflect increasing clinical awareness [8], a broader use of 5-ARIs, or improved pharmacovigilance reporting practices during recent years [16,17].

The current analysis identified signal detections relating to finasteride use and both suicidality (completed suicide ROR, 2.28 [95% CI, 1.26–4.10]) and depression (ROR, 3.76 [95% CI, 3.13–4.51]), whereas dutasteride showed no significant signalling levels for suicidality and depression; this is congruent with similar studies [15]. Indeed, in 2025, the European Medicines Agency (EMA) reported safety concerns about finasteride- and dutasteride-containing medicines, confirming a risk of suicidality as a side effect for finasteride, while the evidence for dutasteride was less robust [18].

Although a definitive pharmacological explanation linking finasteride and dutasteride to depression and suicidality remains elusive, several biologically plausible mechanisms have been proposed. A central hypothesis involves the disruption of neurosteroid synthesis. 5-AR plays a critical role in the conversion of steroids such as progesterone and testosterone into their 5α-reduced metabolites, including DHT and neuroactive steroids such as allopregnanolone. Allopregnanolone is a potent positive allosteric modulator of GABA-A [6,19] receptors and exerts well-established anxiolytic, antidepressant, and stress-buffering effects. Inhibition of 5-AR by finasteride or dutasteride may therefore reduce central nervous system levels of allopregnanolone and related neurosteroids, leading to impaired GABAergic tone and increased vulnerability to anxiety and depressive symptoms [20,21,22].

Beyond neurosteroid depletion, alterations in the androgen and broader sex hormone axis may contribute to psychiatric effects. Finasteride and dutasteride significantly reduce DHT levels while modestly increasing circulating testosterone. Although testosterone itself may have mood-enhancing properties, the imbalance between testosterone and DHT—and downstream effects on androgen receptor signalling—may have complex neuropsychiatric consequences. In particular, DHT has been implicated in modulating sexual function, motivation, and possibly mood regulation. The disruption of androgen signalling may also indirectly affect psychological well-being through sexual adverse effects, including decreased libido, erectile dysfunction, and reduced sexual satisfaction [6]. These effects may contribute to distress, a reduced quality of life, and secondary depressive symptoms, especially in younger individuals [6,23].

Preclinical evidence further supports a role for central nervous system alterations induced by 5-AR inhibition. Animal studies suggest that finasteride can reduce hippocampal neurogenesis, a process closely linked to antidepressant response and mood regulation [19]. Additionally, 5-AR inhibition has been associated with changes in dopaminergic neurotransmission, including reduced dopamine signalling in reward-related brain regions, which may contribute to anhedonia and motivational deficits. Alterations in the expression of steroid receptors and neuroplasticity-related genes have also been reported, indicating that chronic 5-ARI exposure may induce broader neurobiological changes beyond acute neurosteroid depletion [15,20,24].

Emerging evidence also suggests that 5-ARIs may influence stress reactivity and hypothalamic–pituitary–adrenal (HPA) axis function. Neurosteroids such as allopregnanolone play a key role in modulating stress responses, and their depletion may lead to exaggerated stress sensitivity, impaired resilience, and increased susceptibility to mood disorders [21]. This mechanism may be particularly relevant in individuals with a pre-existing vulnerability to psychiatric conditions.

Importantly, epidemiological and pharmacovigilance data indicate that the risk of depression and suicidality may be more pronounced in younger individuals (e.g., <45 years), particularly those using low-dose finasteride for androgenetic alopecia rather than older men treated for benign prostatic hyperplasia. This observation suggests that contextual and psychosocial factors may interact with biological mechanisms. Younger patients may have higher baseline sensitivity to changes in sexual function, body image, and identity-related concerns, which may amplify the psychological impact of adverse effects. Additionally, individuals seeking treatment for hair loss may already experience subclinical distress, potentially increasing their vulnerability to mood disturbances when exposed to neurobiological stressors induced by 5-ARI therapy [14,15,16].

Overall, the available evidence supports a multifactorial model in which neurosteroid depletion, androgen signalling disruption, neuroplastic and neurotransmitter changes, and psychosocial stressors interact to increase the risk of depression and suicidality in susceptible individuals. However, causality remains difficult to establish, and further translational and longitudinal studies are needed to clarify these mechanisms and identify high-risk populations.

Importantly, the relationship between depression and sexual dysfunction is likely bidirectional. Evidence from meta-analytic studies indicates that depressive symptoms increase the risk of sexual dysfunction, while sexual dysfunction itself is a significant predictor of subsequent depression, suggesting a self-reinforcing cycle [25,26]. This reciprocal interaction may partially explain the emergence or worsening of psychiatric adverse events in patients treated with 5α-reductase inhibitors, where drug-induced sexual dysfunction may compound underlying neurobiological vulnerabilities, including dopaminergic alterations.

### 4.1. Psychiatric Assessment of Those Patients Being Treated with Any 5-ARIs

**Figure 3:**
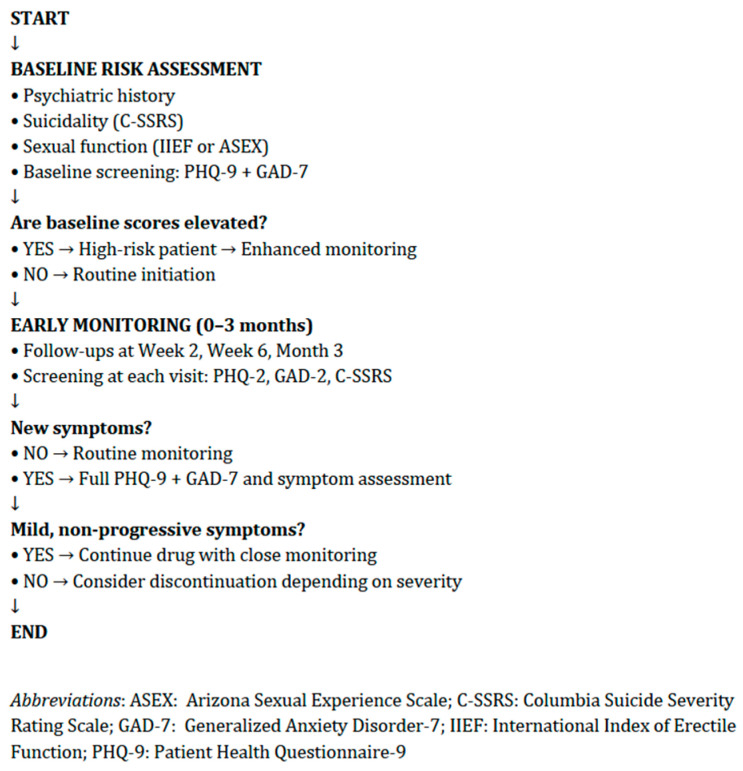
Psychiatric assessment for patients using finasteride/dutasteride; possible evaluating instruments to be considered.

**Figure 4:**
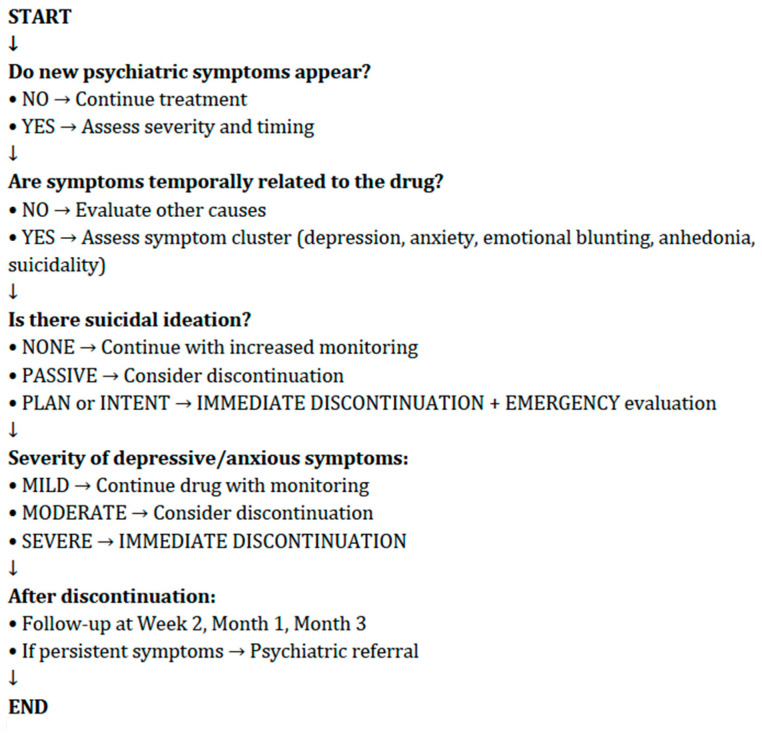
When to Discontinue Finasteride/Dutasteride.

At present, no specific clinical guidelines provide standardized recommendations for the assessment of depression in patients treated with 5ARIs. In Italy, the Agenzia Italiana del Farmaco released a recent Direct Healthcare Professional Communication (DHPC) highlighting the risk of depression and suicidal ideation associated with finasteride and, more cautiously, with dutasteride [27]. This document recommends that patients be informed about potential mood changes and that treatment should be discontinued and medical advice sought if depressive symptoms or suicidal ideation occur. According to the current findings, a specific assessment is required before initiating treatment with any 5-ARIs (Figure 3 and Figure 4). A baseline evaluation should include a comprehensive psychiatric history—for example, previous depression, anxiety, bipolar disorder, past suicide attempts or suicidal ideation, a history of sexual dysfunction and its emotional impact, family psychiatric history, substance use (e.g., alcohol, cannabis, stimulants), as well as an assessment of psychosocial stressors and body-image concerns (particularly relevant in younger patients with alopecia).

Risk factors may include age <45 years, a history of mood disorders, significant body-image concerns, prior severe sexual dysfunction, social isolation, or recent major life stressors. Features that should be monitored regularly include new-onset irritability, anxiety, anhedonia, sleep disturbances, loss of libido or erectile changes, sudden social withdrawal, and passive suicidal thoughts. If such symptoms emerge, a possible temporal relationship with treatment should be assessed, including whether symptoms worsen with dose increases. Discontinuation of the drug should be considered if moderate–severe depression or suicidal ideation develops, or if distressing sexual dysfunction persists and negatively affects mood.

### 4.2. Limitations

As this is a pharmacovigilance study, the data should be interpreted considering several important limitations. Because pharmacovigilance studies rely largely on voluntary spontaneous reports, they are subject to substantial under-reporting levels and various forms of reporting bias, including notoriety and media influence. The absence of denominator data prevents the calculation of true incidence rates, and the quality of individual case reports is often variable, with missing clinical details that limit the assessment of causality. Confounding factors such as comorbidities, disease severity, and polypharmacy are frequently unmeasured, and duplicate or incomplete reports can distort signal detection. Other limitations include the lack of dose information, treatment duration, treatment indication (e.g., prostatic hyperplasia vs. alopecia), and psychiatric history. Selection bias toward more severe or unusual events reduces generalizability, especially for under-represented populations such as children, older adults, and pregnant individuals. Newly marketed drugs may also suffer from insufficient early data, delaying the identification of rare or long-latency adverse reactions. Finally, methodological and regulatory differences across reporting systems and countries introduce heterogeneity that complicates the interpretation of safety signals.

## 5. Conclusions

The present pharmacovigilance study confirms that the use of 5-ARIs is associated with safety signals related to depression, anxiety, suicidality, and sexual dysfunction, with finasteride appearing significantly more prone than dutasteride to generate such signals. The emergence of consistent neuropsychiatric and sexual adverse event signals underscores the importance of a baseline mental health assessment and the close monitoring of patients treated with 5-ARIs, particularly in individuals with prior mood, anxiety, or sexual health concerns. Continued active surveillance and controlled epidemiological studies are needed to determine the true magnitude and clinical significance of these associations.

## Figures and Tables

**Table 1:** Table summarizing the alphabetical representations pertaining to Equations (1) and (2). ‘a’ is the number of reports associated with the drug of interest (e.g., finasteride), ‘b’ is the number of reports not mentioning the event of interest associated with the drug of interest (e.g., all reports minus a), ‘c’ is the number of reports associated with the event of interest relating to the comparator drug (e.g., dutasteride) and ‘d’ is the number of reports for the comparator drug with no mention of the event of interest (e.g., all reports minus c).

	Number of Reports with the Event of Interest	Number of Reports Without the Event of Interest
Drug of interest	a	b
Comparator drug	c	d

**Table 2:** Information regarding Psychiatric ADRs related to Finasteride and Dutasteride retrieved from the FAERS dataset.

Anxiety-Related ADRs			Libido-Related ADRs			Suicide-Related ADRs			Depressive-Related ADRs			
Country	Outcome	Age Range (Years)	Country	Outcome	Age Range (Years)	Country	Outcome	Age Range (Years)	Country	Outcome	Age Range (Years)	Total No. ADRs
GB = 350 CA = 31 NS = 176 NL = 9 IT = 56 US = 672 ES = 40	Other Outcomes = 910 Disabled = 846 Life Threatening = 123 Hospitalized = 272 Required Intervention = 34 Died = 49 Congenital Anomaly = 1	0–5 = 0 6–12 = 0 13–18 = 10 19–64 = 743 65–80 = 25 >81 = 3	GB = 442 US = 788 NS= 390 CA = 43 DE = 82 ES = 49 IT = 110 AU = 15 FR = 74	Other Outcomes = 1299 Disabled = 1140 Required Intervention = 89 Hospitalized = 301 Life Threatening = 154 Congenital Anomaly = 15 Died = 53	0–5 = 0 6–12 = 0 13–18 = 17 19–64 = 1218 65–80 = 53 >81 = 0	GB = 370 DE = 72 FR = 69 NL = 28 CA = 41 NS = 119 ES = 58 AU = 8 US = 197 IT = 27	Other Outcomes = 841 Life Threatening = 268 Hospitalized = 178 Disabled = 384 Died = 144 Congenital Anomaly = 11 Required Intervention = 29	0–5 = 0 6–12 = 0 13–18 = 15 19–64 = 658 65–80 = 47 >81 = 9	GB = 645 DE = 121 NS = 289 CA = 54 NL = 23 AU = 12 FR = 84 IT = 72 ES = 53 US = 738	Other Outcomes = 1494 Disabled = 1032 Life Threatening = 244 Hospitalized = 360 Required Intervention = 64 Congenital Anomaly = 11 Died = 78	0–5 = 0 6–12 = 0 13–18 = 22 19–64 = 1157 65–80 = 89 >81 = 25	**Finasteride (n = 4132)**
US = 5 CA = 5 ES = 2 KR = 1 GB = 1 PT = 1 FR = 1 CH = 1 NL = 1	Disabled = 6 Required intervention = 1 Hospitalized = 4 Life threatening = 5 Other = 20	0–5 = 2 6–12 = 0 13–18 = 0 19–64 = 16 65–80 = 7 >81 = 0	US = 10 NS = 6 ES = 4 GB = 4 CA = 3 FR =3 JP = 2 NL = 1 DE = 1 JM = 1 KR = 1 IT = 1 AU = 1	Disabled = 14 Required intervention = 1 Hospitalized = 3 Life threatening = 2 Other = 28 Congenital anomaly = 3	0–5 = 1 6–12 = 0 13–18 = 0 19–64 = 24 65–80 = 16 >81 = 2	NS = 3 ES = 2 CA = 2 NL = 4 JP = 4 US = 7 GB = 1	Disabled = 6 Required intervention = 2 Hospitalized = 2 Life threatening = 1 Other = 18 Died = 3	0–5 = 1 6–12 = 0 13–18 = 0 19–64 = 10 65–80 = 4 >81 = 0	US = 10 CA = 5 GB = 4 ES = 3 JP = 2 KR = 1 IT = 1 FR = 1 CH = 1	Disabled = 16 Required intervention = 2 Hospitalized = 4 Life threatening = 5 Other = 25	0–5 = 2 6–12 = 0 13–18 = 0 19–64 = 23 65–80 = 13 >81 = 2	**Dutasteride (n = 202)**

Abbreviations: ADR: Adverse Drug Reaction; AU: Austria; CA: Canada; CH: Switzerland; DE: Germany; ES: Spain; FR: France; GB: Great Britain; IT: Italy; JM: Jamaica; JP: Japan; KR: Korea; NL: Nederland; NS: Not Specified; US: United States. Depression-related PTs[MM1.1]: Depressed mood, Depression, Major Depression, Depressive Symptoms, Persistent Depressive Disorder; Suicide-related PTs: Suicidal ideation, Suicide, Completed Suicide, Suicide Attempt, Suicide Threat, Suspected Suicide, Suicidal Behaviour; Libido-related PTs: Loss of libido, libido decreased; Anxiety-related PTs: Anxiety, Generalized Anxiety Disorder, Anxiety Disorder, Social Anxiety Disorder, Mixed Anxiety and Depression, Illness Anxiety Disorder, Adjustment disorder with Anxiety, Adjustment disorder with Anxiety and depression, Anxiety disorder due to a medical condition.

**Table 3:** Reporting Odds Ratio values.

Reaction Group	ROR Finasteride vs. Dutasteride (EXP Upper–Lower 95% CI)
Depression-related	**3.20 (2.33–4.39)**
Suicide-related	**2.72 (1.77–4.19)**
Libido-related	**2.68 (1.96–3.67)**
Anxiety-related	**3.79 (2.51–5.71)**

**Table 4:** Reporting Odds Ratio values.

		ROR (95% CI) Finasteride vs. Dutasteride							
Reaction Group	Age Group	1993–1997	1998–2002	2003–2007	2008–2012	2013–2017	2018–2022	2023–2025	Total
Depressive-related	Under 45	N/A	N/A	N/A	N/A	0.75 (0.17–3.40)	0.47 (0.09–2.57)	2.63 (0.67–10.26)	1.30 (0.59–2.84)
	Over 45	N/A	N/A	1.33 (0.29–6.21)	7.17 (1.62–31.84)	1.55 (0.67–3.56)	2.86 (0.97–8.44)	0.83 (0.23–3.03)	2.60 (1.67–4.06)
	Total	N/A	N/A	1.88 (0.55–6.36)	12.66 (3.00–53.32)	1.46 (0.76– 2.84)	1.53 (0.66–3.58)	1.41 (0.58–3.45)	2.42 (1.67–3.51)
Anxiety-related	Under 45	N/A	N/A	N/A	0.5 (0.03–8.07)	0.46 (0.10–2.08)	0.58 (0.12–2.90)	2.18 (0.46–10.38)	0.94 (0.42–2.11)
	Over 45	N/A	N/A	0	1.28 (0.39–4.17)	2.09 (0.56–7.82)	1.55 (0.34–7.09)	0.69 (0.17–2.76)	1.29 (0.73–2.26)
	Total	N/A	N/A	0.39 (0.09–1.73)	2.59 (0.98–6.80)	1.77 (0.72–4.35)	1.74 (0.64–4.74)	1.41 (0.51–3.93)	1.96 (1.26–3.04)
Suicide-related	Under 45	N/A	N/A	N/A	N/A	0.87 (0.17–4.56)	2.10 (0.24–18.04)	2.37 (0.50–11.25)	1.64 (0.62–4.37)
	Over 45	N/A	N/A	0	0.52 (0.08–3.22)	4.75 (1.04–21.71)	7.94 (1.03–61.20)	1.21 (0.30–4.82)	3.44 (1.76–6.73)
	Total	N/A	N/A	0.42 (0.06–3.15)	2.33 (0.54–9.99)	3.05 (1.06–8.77)	4.56 (1.06–19.60)	1.72 (0.61–4.78)	3.21 (1.87–5.52)
Libido-related	Under 45	N/A	N/A	N/A	1.92 (0.12–30.95)	6.62 (0.79–55.60)	0.23 (0.03–1.93)	0.98 (0.28–3.43)	1.34 (0.62–2.91)
	Over 45	N/A	N/A	2.7 (0.74–9.81)	3.26 (1.41–7.50)	2.19 (0.85–5.64)	0.71 (0.24–2.12)	0.74 (0.19–2.98)	1.28 (0.82–1.98)
	Total	N/A	N/A	2.66 (0.92–7.63)	4.12 (1.98–8.57)	3.90 (1.76–8.68)	1.13 (0.49–2.61)	1.13 (0.46–2.78)	2.17 (1.51–3.12)

Highlight ROR > 1 and CI > 1.

## Data Availability

The FDA Adverse Event Reporting System (FAERS) data is publicly available through the FAERS Public Dashboard at https://www.fda.gov/drugs/fdas-adverse-event-reporting-system-faers/fda-adverse-event-reporting-system-faers-public-dashboard (accessed on 1 April 2026).

## Supplementary Materials

The following supporting information can be downloaded at: https://www.mdpi.com/article/10.3390/brainsci16040394/s1, Figure S1. Finasteride cases deduplication flow chart; Figure S2. Dutasteride cases deduplication flow chart.

## Abbreviations

The following abbreviations are used in this manuscript:

5-ARIs	5α-reductase inhibitors
DHT	Dihydrotestosterone
RORs	Reporting Odds Ratios
FAERS	FDA’s Adverse Event Reporting System
BPH	Benign prostatic hyperplasia
AGA	Androgenic alopecia
ADRs	Adverse drug reactions
GABA	γ-aminobutyric acid
CI	Confidence interval
EMA	European Medicines Agency
