# Supplementary material for: Signal Detection of Depression and Suicidality Associated with Finasteride and Dutasteride: Updated Pharmacovigilance Evidence and Recommendations for Comprehensive Psychiatric Assessment
Source: Brain Sci. 2026 Apr 4;16(4):394. doi: 10.3390/brainsci16040394 (PMC13114726; doi:10.3390/brainsci16040394)
Supplement: Supplementary file 1 [file brainsci-16-00394-s001.zip › brainsci-4168524-supplementary.pdf]

1.List of Preferred Terms (PT) selected for the analysis according to MedDRA

"Depression",

"Anxiety",

"Libido Decreased",

"Loss Of Libido",

"Insomnia",

"Suicidal Ideation",

"Anhedonia",

"Panic Attack",

"Depressed Mood",

"Sleep Disorder",

"Completed Suicide",

"Confusional State",

"Apathy",

"Mental Disorder",

"Stress",

"Emotional Distress",

"Sleep Disorder Due To A General Medical Condition",

"Emotional Disorder",

"Anorgasmia",

"Irritability",

"Mood Swings",

"Major Depression",

"Orgasm Abnormal",

"Mood Altered",

"Anger",

"Suicide Attempt",

"Depression Suicidal",

"Male Orgasmic Disorder",

"Thinking Abnormal",

"Aggression",

"Libido Disorder",

"Agitation",

"Nervousness",

"Hallucination",

"Drug Abuse",

"Premature Ejaculation",

"Psychiatric Symptom",

"Decreased Interest",

"Orgasmic Sensation Decreased",

"Poor Quality Sleep",

"Disturbance In Sexual Arousal",

"Attention Deficit Hyperactivity Disorder",

"Social Avoidant Behaviour",

"Disorientation",

"Affective Disorder",

"Bradyphrenia",

"Psychotic Disorder",

"Middle Insomnia",

"Restlessness",

"Personality Change",

"Obsessive-Compulsive Disorder",

"Emotional Poverty",

"Depersonalisation/Derealisation Disorder",

"Abnormal Dreams",

"Listless",

"Hallucination, Visual",

"Nightmare",

"Panic Disorder",

"Generalised Anxiety Disorder",

"Self Esteem Decreased",

"Anxiety Disorder",

"Derealisation",

"Drug Dependence",

"Somatic Symptom Disorder",

"Personality Disorder",

"Fear",

"Panic Reaction",

"Dissociation",

"Depressive Symptom",

"Libido Increased",

"Social Anxiety Disorder",

"Bipolar Disorder",

"Paranoia",

"Post-Traumatic Stress Disorder",

"Suicidal Behaviour",

"Blunted Affect",

"Mixed Anxiety And Depressive Disorder",

"Flat Affect",

"Psychosexual Disorder",

"Intentional Self-Injury",

"Abnormal Behaviour",

"Initial Insomnia",

"Alcohol Abuse",

"Tension",

"Negative Thoughts",

"Affect Lability",

"Persistent Depressive Disorder",

"Tearfulness",

"Illusion",

"Delirium",

"Phobia",

"Mania",

"Self-Injurious Ideation",

"Frustration Tolerance Decreased",

"Behaviour Disorder",

"Intrusive Thoughts",

"Disturbance In Social Behaviour",

"Adjustment Disorder",

"Obsessive Thoughts",

"Eating Disorder",

"Mental Status Changes",

"Alcoholism",

"Schizophrenia",

"Hallucination, Auditory",

"Reading Disorder",

"Disorganised Speech",

"Bipolar I Disorder",

"Dysphemia",

"Mental Fatigue",

"Body Dysmorphic Disorder",

"Delusion",

"Substance Abuse",

"Borderline Personality Disorder",

"Feeling Of Despair",

"Feelings Of Worthlessness",

"Agoraphobia",

"Suicide Threat",

"Dependence",

"Enuresis",

"Learning Disability",

"Illness Anxiety Disorder",

"Impulsive Behaviour",

"Executive Dysfunction",

"Dysphoria",

"Catastrophic Reaction",

"Self-Destructive Behaviour",

"Communication Disorder",

"Hypersexuality",

"Tic",

"Sopor",

"Sexually Inappropriate Behaviour",

"Obsessive-Compulsive Personality Disorder",

"Psychogenic Erectile Dysfunction",

"Adjustment Disorder With Depressed Mood",

"Bruxism",

"Homicidal Ideation",

"Abulia",

"Autism Spectrum Disorder",

"Gender Dysphoria",

"Tachyphrenia",

"Suspected Suicide",

"Euphoric Mood",

"Impatience",

"Violence-Related Symptom",

"Seasonal Affective Disorder",

"Helplessness",

"Fear Of Crowded Places",

"Antisocial Behaviour",

"Distractibility",

"Rapid Eye Movements Sleep Abnormal",

"Learning Disorder",

"Morose",

"Stereotypy",

"Grief Reaction",

"Dependent Personality Disorder",

"Adjustment Disorder With Mixed Anxiety And Depressed Mood",

"Acute Stress Disorder",

"Psychophysiologic Insomnia",

"Thought Blocking",

"Feeling Guilty",

"Alcohol Withdrawal Syndrome",

"Terminal Insomnia",

"Transient Psychosis",

"Impaired Reasoning",

"Neurosis",

"Indifference",

"Illogical Thinking",

"Performance Fear",

"Sexual Inhibition",

"Drug Use Disorder",

"Substance Dependence",

"Fear Of Death",

"Sleep Disorder Due To General Medical Condition, Insomnia Type",

"Discouragement",

"Hostility",

"Laziness",

"Staring",

"Psychological Trauma",

"Burnout Syndrome",

"Bipolar Ii Disorder",

"Claustrophobia",

"Impulse-Control Disorder",

"Excessive Masturbation",

"Suspiciousness",

"Obsessive Rumination",

"Narcissistic Personality Disorder",

"Loss Of Dreaming",

"Near Death Experience",

"Conversion Disorder",

"Hypomania",

"Mood Disorder Due To A General Medical Condition",

"Acute Psychosis",

"Phonophobia",

"Nicotine Dependence",

"Substance-Induced Psychotic Disorder",

"Disinhibition",

"Tobacco Abuse",

"Delusional Perception",

"Social Fear",

"Inappropriate Affect",

"Negativism",

"Schizoaffective Disorder",

"Factitious Disorder",

"Delusional Disorder, Unspecified Type",

"Merycism",

"Dissociative Disorder",

"Acrophobia",

"Regressive Behaviour",

"Lack Of Empathy",

"Alcohol Problem",

"Delusional Disorder, Somatic Type",

"Thermophobia",

"Sleep Terror",

"Obsessive-Compulsive Symptom",

"Psychomotor Retardation",

"Hallucination, Tactile",

"Morbid Thoughts",

"Gambling Disorder",

"Daydreaming",

"Catatonia",

"Psychotic Symptom",

"Somnambulism",

"Selective Eating Disorder",

"Cyclothymic Disorder",

"Dissociative Identity Disorder",

"Neuropsychological Symptoms",

"Poverty Of Speech",

"Trance",

"Delirium Tremens",

"Persecutory Delusion",

"Deja Vu",

"Bulimia Nervosa",

"Aversion",

"Grandiosity",

"Adjustment Disorder With Anxiety",

"Poriomania",

"Soliloquy",

"Time Perception Altered",

"Delusional Disorder, Persecutory Type",

"Lack Of Spontaneous Speech",

"Dyssomnia",

"Paranoid Personality Disorder",

"Schizoaffective Disorder Bipolar Type",

"Organic Brain Syndrome",

"Anxiety Disorder Due To A General Medical Condition",

"Change In Sustained Attention",

"Aerophobia",

"Perseveration",

"Alcoholic Hangover",

"Self Esteem Inflated",

"Coprolalia",

"Exploding Head Syndrome",

"Constricted Affect",

"Somniphobia",

"Loose Associations",

"Poverty Of Thought Content",

"Schizoid Personality Disorder",

"Psychological Factor Affecting Medical Condition",

"Shared Psychotic Disorder",

"Reactive Psychosis",

"Adjustment Disorder With Mixed Disturbance Of Emotion And Conduct",

"Substance-Induced Mood Disorder",

"Psychogenic Movement Disorder",

"Somatoform Genitourinary Disorder"

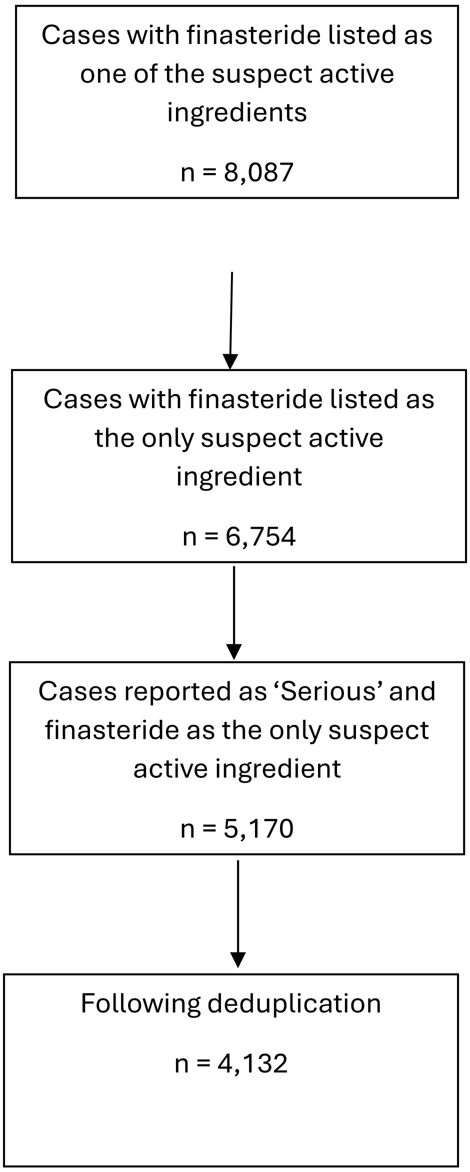

Figure S1. Finasteride cases deduplication flow chart

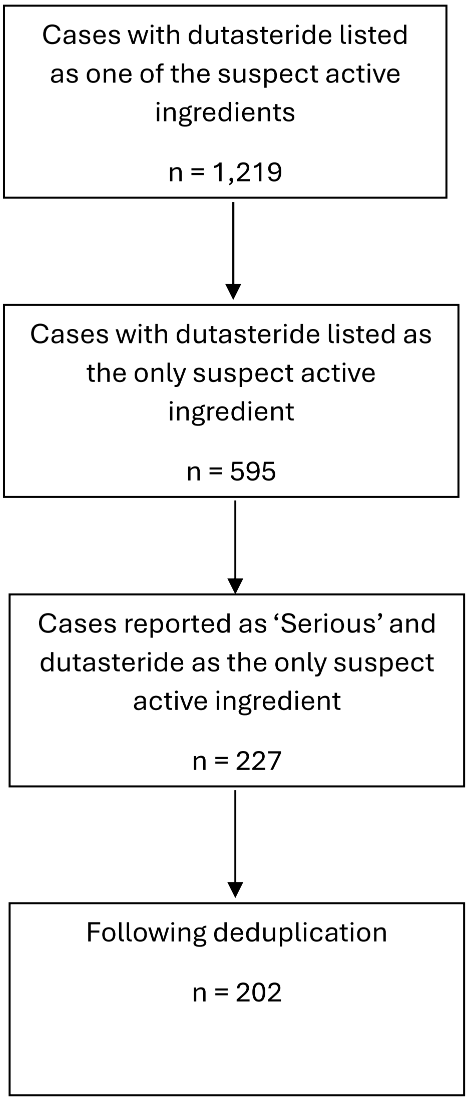

Figure S2. Dutasteride cases deduplication flow chart
